# Opposing Forced Displacement and Nuclear War is a Professional Obligation for Healthcare Workers and Public Health Advocates

**DOI:** 10.7759/cureus.88431

**Published:** 2025-07-21

**Authors:** Bilal Irfan, Muhammad Hamza Shah

**Affiliations:** 1 Center for Surgery and Public Health, Brigham and Women's Hospital, Boston, USA; 2 Michigan Alzheimer's Disease Center, University of Michigan, Ann Arbor, USA; 3 Center for Bioethics, Harvard Medical School, Boston, USA; 4 Centre for Anatomy, The University of Edinburgh, Edinburgh, GBR; 5 School of Medicine, Queen's University Belfast, Belfast, GBR

**Keywords:** armed conflict, ethics, forced displacement, nuclear war, professional obligation

## Abstract

Forced displacement and the looming threat of nuclear conflict are now among the most potent, preventable drivers of global morbidity and mortality. This editorial argues that health professionals, bound by ethical mandates to promote and preserve life, bear a positive duty not only to treat the downstream consequences of war but to confront the policies that generate them. We outline the cascading public-health harms that accompany mass displacement, including communicable-disease outbreaks, interruption of chronic care, gender-based violence, and intergenerational trauma. We contend that silence in the face of such structural violence constitutes professional complicity. The piece therefore calls on healthcare workers, public health institutions, and professional societies to produce and disseminate rigorous evidence on war-related health impacts, advocate for the cessation of hostilities and safe humanitarian corridors, refuse collaborations that militarize health systems, and embed peace-medicine and humanitarian-law training into their curricula. Healthcare workers can utilize academic spaces, including conferences, journals, and lectures, to speak, document, and share the realities of the consequences of war and its impact on population health. Neutrality, we suggest, must align with patients, not with powers perpetuating displacement and existential nuclear peril.

## Editorial

The sight of patients uprooted from homes, jostled across borders, or sheltering beneath shards of ruined hospitals is no longer an aberration: it is the epidemiological signature of modern war. As a global collective of healthcare workers and public health advocates, we may claim lineage with Hippocrates and Nightingale; yet, the pledge to preserve life is daily subverted by state policies that manufacture mass displacement. Our professional conscience, therefore, demands more than individual acts of care [1]. It obliges resolute opposition to the engines of violence that create the very diseases and injuries we are asked to treat.

The scale of forced displacement is staggering. There are approximately 123.2 million people forcibly displaced right now, be it living as refugees, asylum-seekers, internally displaced, or another status, at the close of 2024 [2]. Epidemiologists know that precarious shelter, food insecurity, interrupted immunization, chronic stress, and exposure to gender-based violence combine to erode life expectancy; clinicians see the downstream fevers, fractures, and psychoses. The first obligation of prevention is to confront and dismantle structures that drive forced displacement and systematically erode the foundations of public health.

Yet conflicts continue to widen the gyre. In just the past three years alone, millions across Ukraine, Gaza, Sudan, Lebanon, Syria, Iran, and Yemen have been temporarily or permanently displaced, and thousands across the world continue to flee armed violence directed at civilians. These theatres share some etiological features: indiscriminate use of weaponry, obstruction of humanitarian corridors, and political narratives that dehumanize civilian populations. 

Other flashpoints now threaten a catastrophic leap from conventional to nuclear war, a scenario whose public-health ramifications are literally incalculable. In May 2025, nuclear-armed India and Pakistan exchanged cross-border strikes. Barely a month later, Israeli and American aircraft struck Iran’s nuclear facilities. Such incidents edged South Asia and the Middle East toward a threshold where no humanitarian response would suffice. Recognizing this reality, the World Health Assembly adopted a resolution directing the World Health Organization (WHO) to renew its dormant program on the health effects of nuclear war and to report on the existential threat such weapons pose to global health and survival [3].

If war and displacement are the points of exposure, then health workers are duty-bound to mitigate and, where possible, eliminate them. The normative foundations are unambiguous. Our norms must consider moving beyond safeguarding the physician’s right to treat, a positive obligation to oppose policies that produce illnesses and forced displacement is vital. Silence in the face of systematic violence is, ethically, complicity.

A public-health lens strengthens that imperative. Forced displacement through war propagates communicable disease by fracturing the process of care, foregoing routine immunizations, and crowding families into areas of resource scarcity. It multiplies non-communicable morbidity as the hypertension drugs run out. It increases mortality when war strategies involve bombing dialysis units and hospitals. It seeds transgenerational trauma, with epigenetic imprints already demonstrated in children born in exile. To frame opposition to such policies as extraneous “politics” is therefore to misunderstand the object of our science: populations. Advocacy to mitigate armed conflict is not mission creep; it is disease prevention at its most fundamental level.

What, then, should the profession do? First, we must generate and disseminate rigorous evidence on the health impacts of displacement. Secondly, our professional associations must wield that evidence in policy arenas, demanding a just cessation of hostilities, safe corridors for refugees, and compliance with international treaties and obligations. Third, we must refuse partnerships that normalize the militarization of health systems or allow their robustness to be leveraged as negotiable points. Medical and public health curricula should incorporate peace medicine, humanitarian law, and advocacy skills, preparing practitioners to navigate the moral terrain of modern conflict. The ethical mandate to resist forced displacement and call for the prosecution of war crimes arises directly from the core commitments that define the health professions. Beneficence obliges clinicians to pursue the well‑being of persons under their care, but contemporary practice has demonstrated that among the gravest threats to health are those often scripted far upstream of the clinical encounter, in policies that weaponize war, deliberate starvation, ethnic cleansing, or indiscriminate bombardment. Professional charters that cite codes stemming from the Declaration of Geneva or nursing guidelines, when intersecting with conflict medicine, at times already affirm that a practitioner’s primary loyalty is to the health and well‑being of patients; once that loyalty is taken seriously, it inevitably extends to opposing state or non‑state actions that destroy hospitals, sever supply chains, criminalize humanitarian corridors, or permanently exile vulnerable communities. Justice and respect for human dignity deepen the claim: when health workers possess both frontline witness and societal credibility, their silence becomes an enabling condition for structural violence. Advocacy is, therefore, not an optional add‑on to clinical excellence, nor is it a partisan diversion; it is the forward‑looking expression of non‑maleficence and the social contract that grants the professions their autonomy. Accepting the epistemic privilege of seeing a bullet’s trajectory from policy to body confers a correlative duty to raise the alarm, document violations, and demand redress within the frameworks of international humanitarian and human rights law. Transforming that duty into practice requires a continuum of advocacy forums that align with the diverse roles clinicians inhabit. At the level of individual agency, practitioners can record injury patterns, certify causes of death in language that courts recognize, and ensure displaced patients receive continuity of care through interoperable electronic records. At the institutional level, specialty societies can pass binding resolutions that condition conference sponsorships and research collaborations on adherence to humanitarian norms. At the same time, academic journals can expedite the publication of evidence on the public health sequelae of displacement to reorient donor priorities. System‑level activism draws on the collective capacities of professional associations, patient advocacy organizations, and global alliances of medical, nursing, and public‑health bodies; together they can share information in international tribunals, provide expert affidavits for sanctions hearings, lobby ministries to suspend arms exports that target health infrastructure, and design curricula that teach liberation medicine alongside trauma surgery. Digital platforms enable diaspora clinicians to reach a wide audience, and transnational research networks can generate data that disproves official denials of atrocity. In each arena, the operative ethical concepts can remain constant, be it beneficence, justice, or solidarity, but the modalities of action are calibrated to the practitioner’s scope of control and risk exposure. When viewed through this tiered architecture, opposition to forced displacement and war crimes is not a rhetorical flourish: it is a structured, feasible extension of professional responsibility, anchored in existing codes, empowered by collective mechanisms, and indispensable to the prevention of war crimes and the preservation of human life. To mount such advocacy with integrity, the profession must also consider interrogating its own governance. Hospitals and academic centers that derive tax exemptions from their putative community benefit cannot claim solidarity with the displaced while their governing boards are stacked with private‑equity financiers, health‑insurance magnates, or weapons‑industry chiefs whose business models feed the very conflicts that uproot patients [4]. When directors who profit from discriminatory lending, predatory rent extraction, or arms sales decide capital budgets and charity‑care policies, the institution’s fiduciary compass tilts toward asset protection, rather than deterrence of violence [5]. Reclaiming board seats for frontline clinicians, migrant‑justice organizers, and war‑zone public health experts would align strategic planning with the ethical duties articulated above, transforming hospitals from passive repositories of ventilators into civic moral agents capable of opposing displacement at its geopolitical roots. Such governance reform is itself a form of preventive medicine: by severing institutional dependence on war‑profiteering and extractive finance, health systems can help dismantle the economic incentives that perpetuate forced migration and edge the world toward nuclear catastrophe.

Critics may object that taking sides jeopardizes neutrality. The answer is that neutrality is not value-free. Displaced people face hazards long before a bullet pierces flesh; our neutrality must lie with the patient, not the power that uproots them. By documenting atrocities, speaking truth to power, and refusing to be co-opted, we can embody the highest tradition of the healer.

Conclusion

The stakes could not be higher. Nuclear firestorms would generate soot sufficient to collapse global agriculture, plunging billions into famine and reversing centuries of health progress in a single season. Preventing such an outcome is important, just as is preventing the indiscriminate shelling that drives a family to seek shelter in another place. The continuum of violence ends, if unchecked, in collective annihilation.

Survival is the first and foremost prescription. Yet the patient list has swelled to include tens of millions of forcibly displaced people and an entire planet under nuclear Damocles. To remain faithful to our calling, health professionals must use their professional instruments, be it epidemiological data, clinical testimony, or moral authority, to oppose unrestricted war and the policies of forcible displacement that follow in its wake. Anything less abdicates the duty we owe to those who have already lost homes, limbs, and kin, and to generations yet unborn whose health depends on the peace we secure today.
